# Immunogenic Comparison of Nucleic Acid-Based Vaccines Administered by Pyro-Drive Jet Injector

**DOI:** 10.3390/vaccines12070757

**Published:** 2024-07-09

**Authors:** Jiayu A. Tai, Tomoyuki Nishikawa, Hiroki Hayashi, Yu-Diao Kuan, Kunihiko Yamashita, Hironori Nakagami

**Affiliations:** 1 Department of Device Application for Molecular Therapeutics, Graduate School of Medicine, Osaka University, 2-2 Yamada-oka, Suita 565-0871, Osaka, Japan; annatai@impulse.med.osaka-u.ac.jp (J.A.T.); tomonishi@gts.med.osaka-u.ac.jp (T.N.); ydkuan@impulse.med.osaka-u.ac.jp (Y.-D.K.); ku_yamashita@impulse.med.osaka-u.ac.jp (K.Y.); 2 Department of Health Development and Medicine, Graduate School of Medicine, Osaka University, 2-2 Yamada-oka, Suita 565-0871, Osaka, Japan; hayashih@cgt.med.osaka-u-ac.jp; 3 Medical Device Division, Life Sciences Strategic Business Unit, Daicel Corporation, 2-2 Yamada-oka, Suita 565-0871, Osaka, Japan; 4 Center for Infectious Disease Education and Research (CiDER), Osaka University, 2-2 Yamada-oka, Suita 565-0871, Osaka, Japan

**Keywords:** DNA vaccine, mRNA vaccine, needle-free injector

## Abstract

mRNA vaccines were successfully developed and approved for emergency use to fight coronavirus disease 2019. However, the effect of DNA vaccines against SARS-CoV-2 is considerably lower than that of mRNA vaccines. A pyro-drive jet injector (PJI) efficiently delivers plasmid DNA intradermally into animal models. Here, we compared the immunogenic potential of DNA and mRNA vaccines in mice using the same platform. PJI was used to deliver naked mRNA and pDNA and their efficacy in inducing antigen expression and immune responses was assessed. Our results showed that PJI efficiently delivered mRNA into the skin, and a smaller effective dose than that of pDNA injection was required to achieve similar levels of antigen expression. The PJI-delivered CpG-free pDNA vaccine efficiently induced antigen-specific antibody production and a cell-mediated IFN-γ response compared to the mRNA vaccine, as well as the upregulation of inflammatory cytokines (IL-6, IFN-γ, and IL-1β) in the skin and lymph nodes. However, the intradermal mRNA vaccine was significantly less immunogenic than the standard intramuscular mRNA-lipid nanoparticle vaccine, despite equivalent mRNA dosages. Improvements in lipid nanoparticle and mRNA technology have revolutionized mRNA vaccines, and DNA vaccines can be similarly modified for higher clinical efficacy.

## 1. Introduction

The first case of coronavirus disease 2019 (COVID-19) was reported in Wuhan, China in December 2019, spreading worldwide at an alarming rate and becoming an unprecedented pandemic [1]. The early availability of a rapidly available vaccine will help reduce the morbidity rate of high-risk individuals, such as frontline medical staff.

Inactivated vaccines have been commonly used in infectious diseases [2]; however, in the recent SARS-CoV-2 pandemic, nucleic acid-based vaccines using mRNA or plasmid DNA (pDNA) were rapidly developed as emergency-use vaccines, utilizing novel gene therapy technologies for vaccine development [3]. Surprisingly, the mRNA vaccines developed by Pfizer/BioNTech and Moderna were developed within a year and have shown a high impact in clinical trials [4,5]. DNA vaccines were also rapidly developed, and a vaccine developed by Zydus Cadila (ZyCoV-D) received rapid approval in India [6]. However, the effect of DNA vaccines on SARS-CoV-2 infection is not as prominent as that of the mRNA vaccines. Despite encouraging preclinical data, most DNA vaccines in the vaccine pipeline have not surpassed mRNA vaccine efficacy in clinical trials [7,8,9].

In the history of DNA vaccines, more than a hundred clinical trials that focus on DNA vaccination have been registered, but most of them were not successful up to now. Nevertheless, some DNA-based vaccines were approved for veterinary use, including a vaccine against West Nile virus in horses [10], and melanoma in canines [11]. The main unsolved issue is the low immunogenicity of DNA vaccines in humans, potentially because of the low amount of antigen expression or weak innate immune response. To overcome this issue, DNA delivery and transfection systems have been optimized over many years [12]. In fact, jet injectors [13] and electroporation [14] have enhanced vaccine responses through an increased efficiency of DNA delivery and have been recently utilized in clinical trials.

The pyro-drive jet injector (PJI) is a novel needleless injection device capable of intradermally delivering different pDNA in different animal models [15,16,17,18], and has also been used to intradermally deliver carrier-free mRNA into mouse skin [19]. In this study, to clarify the issues regarding potential immune response differences between DNA and RNA vaccines, we compared the use of both DNA and mRNA vaccines on the same platform in mice, without using lipid nanoparticles (LNPs). We tested the use of PJI to intradermally deliver naked mRNA and pDNA to assess its efficiency in inducing antigen expression and the subsequent antibody- and cell-mediated immune responses. Additionally, we sought to compare and evaluate the effect of RNA vaccine administration using PJI with that of RNA vaccine using LNPs.

## 2. Materials and Methods

### 2.1. Animals

Eight-week-old female BALB/c and C57BL/6NJcl mice (CLEA Japan, Inc., Tokyo, Japan) were housed in a temperature- and light/dark cycle-controlled pathogen-free facility with free access to food and water. All the animals were strictly handled following protocols approved by the Animal Committee of Osaka University Graduate School of Medicine (#03-004-017).

### 2.2. Plasmid DNA and mRNA Vaccines

pcDNA3-OVA was a gift from Sandra Diebold and Martin Zenke (Addgene plasmid #64599; http://n2t.net/addgene:64599; RRID: Addgene_64599; Addgene, Watertown, MA, USA) [20]. pcDNA3-OVA is a CpG-containing mammalian expression vector with a cytomegalovirus (CMV) promoter encoding the ovalbumin (OVA) gene. pCpGfree-OVA and pCpGfree-mcs (InvivoGen, San Diego, CA, USA) are CpG-free mammalian expression vectors that contain the human elongation factor 1α promoter. pCpGfree-OVA contains a CpG-free allele of the OVA gene. pCpGfree-LUC was constructed by inserting the luciferase (LUC) gene isolated from pGL4.49 (Promega Corporation, Madison, WI, USA) into the multiple cloning site of pCpGfree-mcs. OVA and LUC mRNA (catalog#MRNA41, #MRNA12, OZ Biosciences, San Diego, CA, USA) are 5-methoxyuridine (5-moU)-modified mRNAs.

### 2.3. Preparation of mRNA-LNPs

The mRNA-LNPs used here were based on the lipid composition of a clinically approved BNT162b2 formulation [21], and prepared using a generalized protocol collated from multiple sources [22,23]. Briefly, ionizable cationic lipid ALC-0315 (#HY-138170, MedChemExpress LLC, Monmouth Junction, NJ, USA), neutral lipid 1, 2-distearoyl-sn-glycero-3-phosphocholine (DSPC; #850365P, Avanti Polar Lipids, Inc., Birmingham, AL, USA), cholesterol (#C8667, Sigma Aldrich, St. Louis, MO, USA), and PEGylated lipid ALC-0159 (#HY-138300, MedChemExpress) were mixed at a molar ratio of 46.3:9.4:42.7:1.6 mol% in ethanol. OVA mRNA was prepared in 10 mM citrate buffer at a nitrogen/phosphate ratio of 6. Both the solutions were mixed together at a total flow rate of 12 mL/min and a flow rate ratio of 3:1 *v*/*v* (aqueous/organic phase) using the NanoAssemblr Ignite (Precision NanoSystems Inc., Cytiva, Vancouver, BC, Canada). Newly encapsulated mRNA-LNPs were dialyzed and concentrated using 100 K Amicon Ultra centrifugal filters (#UFC/910096, Merck Millipore, Burlington, MA, USA). The LNP particle size and zeta potential were checked using a Zetasizer Nano ZS (Malvern Instruments, Malvern, UK). The encapsulated mRNA content in the mRNA-LNPs was determined using the Quant-iT RiboGreen RNA Reagent and Kit (#11490, Invitrogen, Thermo Fisher Scientific Inc., Darmstadt, Germany). The final mRNA-LNP vaccine was prepared by diluting mRNA-LNP to a final volume of 40 µL, containing 1 µg of mRNA in 10% sucrose solution.

### 2.4. Mouse Vaccination Protocols

For intradermal immunization with pDNA or mRNA, the indicated dose (in a fixed volume of 20 µL) of each vaccine or phosphate-buffered saline (PBS; Nacalai Tesque Inc., Kyoto, Japan) was injected intradermally into the mouse flank regions using PJI (Actranza Lab, Type: Mouse, Daicel Corporation, Osaka, Japan). For post-vaccination immune response analyses, the mice were intradermally injected as described above at weeks 0 and 2, with two vaccinations (prime and boost injections) on the same flank each time. For vaccination using mRNA-LNP, mRNA-LNP containing an equivalent dose of 1 µg OVA mRNA in a total volume of 40 µL was divided into two half-doses of 20 µL and injected intramuscularly into both the left and right thigh muscles of each mouse using a 30G needle syringe for two vaccinations (prime and boost) at weeks 0 and 2.

### 2.5. Detection of OVA Expression by Enzyme-Linked Immunosorbent Assay (ELISA)

To analyze the OVA expression after the OVA pDNA or mRNA vaccination using PJI, the injected skin region was excised with a 5 mm diameter biopsy punch (#Bp-50F, Kai Industries Co., Ltd., Seki, Japan) 24 h after the injection. Total protein was extracted by homogenization using the Fastprep-24 5G system (MP Biomedicals, LLC, Santa Ana, CA, USA). The OVA expression was quantified using an OVA ELISA kit (ITEA Inc., Tokyo, Japan).

### 2.6. ELISA-Based Anti-OVA Antibody Titer Analysis

To determine the antibody-mediated immune response, serum was collected from the immunized mice at weeks 2 and 4. Anti-OVA antibody titer analysis using ELISA was performed as previously described in [15]. The mouse serum samples were diluted from 10- to 31250-fold in a 5% skim milk blocking buffer and incubated in recombinant OVA protein (#015-24731, Wako Pure Chemicals Industries Ltd., Tokyo, Japan) pre-coated ELISA plates overnight at 4 °C. The plates were washed with PBS-Tween 20, labeled with Amersham ECL anti-mouse IgG horseradish peroxidase-linked whole antibody (1:1000 dilution; #NA931, GE Healthcare, Cytiva, Marlborough, MA, USA) for 3 h, washed, and finally detected using 3,3′-5,5′-tetramethylbenzidine (TMB; #T0440, Sigma Aldrich) for 30 min, before neutralization using equal volumes of 0.9 N sulfuric acid. Absorbance was measured at 450 nm using an iMark microplate reader (Bio-Rad Laboratories, Hercules, CA, USA). The average OD_450_ value was applied as follows: the cut-off value was 0.2 (OD_450_) and the antibody titer was equal to the fold dilution at the cut-off point. Antibody titers were calculated using GraphPad Prism 9 (GraphPad Software Inc., San Diego, CA, USA).

### 2.7. Detection of Luciferase Expression in Skin Tissue Samples

To detect the LUC expression following the mRNA injection using PJI at 3, 6, and 24 h after the C57BL/6NJcl mice were injected once with LUC-mRNA (0.04, 0.2, 1.0, or 5.0 µg/20 µL), the injected skin tissues were collected using a 5 mm diameter biopsy punch (Kai Industries Co.). To compare the LUC expression between the mRNA and pDNA injection, LUC-mRNA (0.2 or 1.0 µg/20 µL) and pCpGfree-LUC pDNA (10 or 50 µg/20 µL) were injected intradermally into mouse flanks by PJI, and the injected skin tissue was harvested as described above. The biopsied skin tissues were minced with scissors and lysed using a FastPrep-24 5G homogenizer (#6005-500, MP Biomedicals). The LUC expression was determined using a Luciferase Assay reagent (#E1501, Promega) according to the manufacturer’s instructions. The luminescent activity was measured using a GloMax Discover microplate reader (Promega).

### 2.8. In Vivo Bioluminescence Analysis

To monitor LUC expression over time, LUC-mRNA (0.2 or 1.0 µg/20 µL) was injected into the back flanks of the BALB/c mice via PJI. The mice were then injected with D-luciferin (#eLUCK, GoldBio, St. Louis, MO, USA), and luminescence activity was visualized at 1, 3, 6, 12, 24, and 48 h after mRNA injection using an IVIS Lumina II (Caliper Life Sciences, Hopkinton, MA, USA). Bioluminescence analysis was performed using the Living Image Software version 4.0 (Caliper Life Sciences).

### 2.9. IFN-γ ELISpot Assay

Splenocytes were harvested from the mice immunized twice at five weeks. Splenocytes were prepared as described previously [17]. Mouse IFN-γ ELISpot assay (R&D Systems, Inc., Minneapolis, MN, USA) was performed according to the manufacturer’s instructions. Briefly, the recovered splenocytes (3 × 10^5^ cells/well) were plated on capture antibody-coated 96-well PVDF plates (Merck Millipore) in complete RPMI 1640 medium supplemented with 10% fetal bovine serum (MP Biomedicals), 0.1 mg/mL penicillin/streptomycin, and 50 µM β-mercaptoethanol (Nacalai Tesque) and stimulated in the absence and/or presence of 10 µg/mL recombinant OVA for 48 h at 37 °C in a humidified atmosphere of 5% CO_2_. The membranes were stained using the ELISpot Blue Color Module (R&D Systems), and the number of positively stained spots was counted. The effective IFN-γT cell response for each mouse sample was calculated using the following formula: (total number of spots in OVA-stimulated medium) − (number of spots in no-stimulation medium).

### 2.10. Quantitative Real-Time PCR Analysis

Skin tissue and proximal lymph node samples were collected 24 h after prime or boost injections as described. Total cell lysates were obtained after homogenization using the Fastprep-24 5G system (MP Biomedicals). Total RNA was extracted from all the samples using the Maxwell RSC simplyRNA Tissue Kit (#AS1340, Promega) and Maxwell RSC Instrument (Promega). Total RNA (1 µg) was reverse transcribed into cDNA using the ReverTra Ace qPCR RT kit (#FSQ-201, Toyobo Co., Ltd., Osaka, Japan), and then amplified by quantitative real-time PCR with the QuantStudio 3 real-time PCR system (Applied Biosystems, Thermo Fisher Scientific) using the Real-Time PCR Master Mix (#QPK-101, Toyobo) and TaqMan probes for murine IL-6 (Mm00446190_m1), IFN-γ (Mm01168134_m1), IL-1β (Mm9999906_mH), and β-actin (Mm02619580_g1; all Applied Biosystems, Thermo Fisher Scientific). The expression levels of the target genes were normalized to those of *β-actin*.

### 2.11. Statistical Analysis

All the values are presented as the mean ± standard error of the mean. All the statistical analyses were performed using GraphPad Prism 9 (GraphPad Software). Unpaired Welch’s *t*-tests were used to compare between the two different groups in the initial CpG/CpG-free pDNA OVA expression analysis. The statistical comparison of luciferase activity, antibody titers, and T cell IFN-γ response with three or more groups was performed using a one-way ANOVA test and adjusted for multiple testing using Tukey’s multiple comparisons test. For luciferase detection over time, a two-way ANOVA test was used and adjusted with Šídák’s multiple comparisons test. For quantitative real-time analysis, a two-way ANOVA test was used adjusted with Tukey’s multiple comparison test. Statistical significance was set at *p* < 0.05. * *p* < 0.05, ** *p* < 0.01, *** *p* < 0.001, **** *p* < 0.0001.

## 3. Results

### 3.1. Effects of OVA pDNA Vaccine Delivered by PJI on Protein Expression and Antibody Response

**Figure 1:**
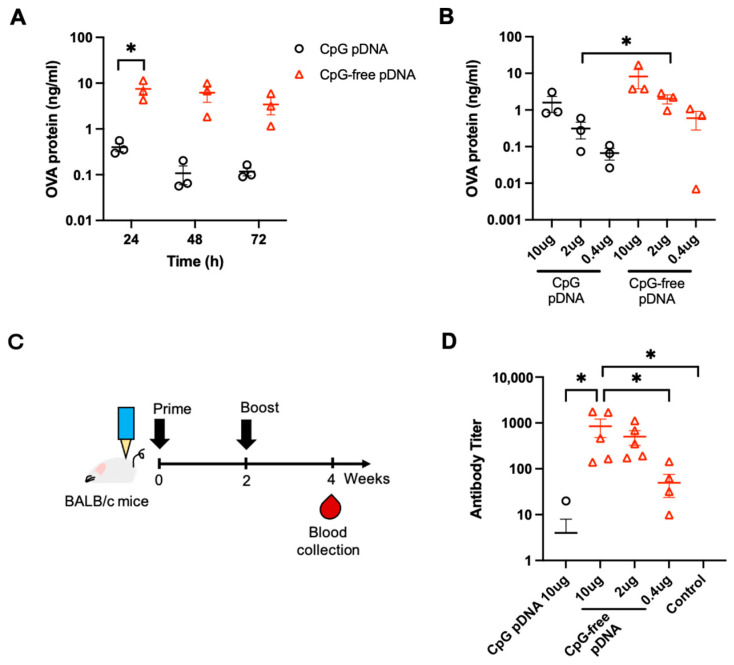
OVA pDNA expression and antibody response after PJI delivery. (**A**) OVA expression at 24, 48, and 72 h after the intradermal injection of the CpG-containing OVA-encoding pDNA (CpG pDNA: 10 µg/20 µL) and the CpG-free OVA-encoding pDNA (CpG-free pDNA: 10 µg/20 µL) into female BALB/c mouse skin by PJI (*n* = 3 in each group). (**B**) OVA protein expression 24 h after the intradermal injection of different OVA pDNA doses (0.4, 2, or 10 µg/20 µL) into BALB/c mouse skin by PJI (*n* = 3 in each group). (**C**) Time course of the experiment. The BALB/c mice were vaccinated twice (prime and boost) intradermally using PJI at a two-week interval and anti-OVA antibody titer was analyzed at two weeks after the last vaccine. (**D**) Anti-OVA antibody titer at 4 weeks after the prime/boost injections of the CpG pDNA (10 µg/20 µL) and CpG-free pDNA (0.4, 2, or 0.4 µg/20 µL) into the BALB/c mouse or non-vaccinated control mouse skin by PJI (*n* = 5 in each group). All the results are presented as the mean ± standard error of the mean (SEM). The *p*-values were analyzed using the unpaired Student’s *t*-test in (**A**,**B**) and one-way ANOVA test adjusted for multiple testing using Tukey’s multiple comparisons test in (**D**). * *p* < 0.05.

It has been suggested that CpG-free DNA may prolong overall gene expression and lower immunogenicity to undesirable innate immune responses [24,25]. To test whether the absence of CpG in the pDNA vaccine could potentially affect OVA expression, 10 µg of CpG-containing OVA-encoding pDNA (pDNA3.1-OVA; CpG pDNA) and 10 µg of CpG-free OVA-encoding pDNA (pCpGfree-OVA; CpG-free pDNA) were intradermally injected into the skin of female BALB/c mice by PJI and analyzed for OVA expression. In fact, the CpG-free pDNA consistently induced higher OVA expression than the CpG pDNA at 24–72 h (Figure 1A). Next, we evaluated OVA expression 24 h after different pDNA doses (0.4 to 10 μg) were administered by PJI and found that not only was OVA expression dose-dependent, but CpG-free pDNA also induced higher OVA expression than CpG pDNA at all three doses tested (Figure 1B). To further evaluate whether CpG-free pDNA affected the vaccine-induced antibody response, the BALB/c mice were vaccinated twice intradermally using PJI at a two-week interval and the anti-OVA antibody titer was analyzed two weeks after the prime/boost vaccine (Figure 1C). The CpG-free pDNA vastly outperformed the CpG pDNA in inducing antibody titer against OVA at equivalent doses of 10 µg (212-fold; Figure 1D). Despite the similar OVA expression between the CpG-free pDNA (2 µg) and CpG pDNA (10 µg, 1.27-fold difference; Figure 1B), the former induced a 126-fold higher anti-OVA antibody titer than the latter (Figure 1D). These results suggested that the CpG-free pDNA can induce strong antigen expression and a robust antibody immune response.

### 3.2. Evaluation of PJI-Delivered mRNA Vaccine on Gene Expression

**Figure 2:**
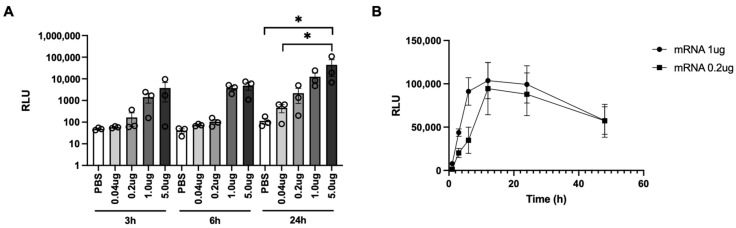
Evaluation of PJI-delivered mRNA vaccine on luciferase activity. (**A**) Luciferase (LUC)-mRNA (0.04, 0.2, 1, or 5 µg/20 µL) or PBS was injected intradermally on C57BL/6NJcl mouse backs by PJI (*n* = 3 in each group), and luciferase activity in the injected skin tissues were analyzed after 3, 6, and 24 h after the injection. (**B**) LUC activity 1, 3, 6, 12, 24, and 48 h after the LUC-mRNA injection (0.2 or 1 µg/20 µL) into the BALB/c mouse backs by PJI measured by in vivo bioluminescence analysis using IVIS (*n* = 3 in each group). All the results are shown as the mean ± SEM. Luciferase activity is represented by relative light units (RLU). The *p*-values were analyzed using the one-way ANOVA test adjusted for multiple testing using Tukey’s multiple comparisons test in (**A**) and two-way ANOVA test adjusted using Šídák’s multiple comparisons test in (**B**). * *p* < 0.05.

In our previous work, we showed the multiple applications of PJI for the intradermal delivery of different pDNA in different animal models [15,16,17]. Modified mRNA vaccines are commonly formulated with LNPs to improve stability and cellular uptake [26] and are usually administered intramuscularly via traditional needle syringes [4,5]. Here, we tested the intradermal delivery of naked 5-moU-modified mRNA using PJI to assess its efficiency in inducing antigen expression and the subsequent antibody- and cell-mediated immune responses via this administration route. First, to test mRNA delivery and dose efficiency, LUC-mRNA (0.04 to 5 µg) was intradermally injected into the backs of C57BL/6NJcl mice by PJI, and luciferase activity in the injected skin tissues was analyzed 3, 6, and 24 h after the injection. Luciferase activity was detected as early as 3 h and was highest at 24 h at each mRNA concentration. The luciferase activity also increased proportionally with the mRNA concentration at each time point, indicating a dose-dependent effect (Figure 2A). To observe the LUC expression over time after mRNA injection by PJI, in vivo real-time bioluminescence analysis was performed at 1, 3, 6, 12, 24, and 48 h after mRNA injection into the skin of the BALB/c mice using IVIS imaging. Regardless of the dose (0.2 or 1 µg), luciferase activity peaked between 12 and 24 h after the injection and gradually decreased until 48 h (Figure 2B). These results show that mRNA can be delivered into the skin by PJI and induce gene expression.

### 3.3. Comparison of PJI-Delivered pDNA and mRNA Vaccines on Gene Expression and Immune Response

**Figure 3:**
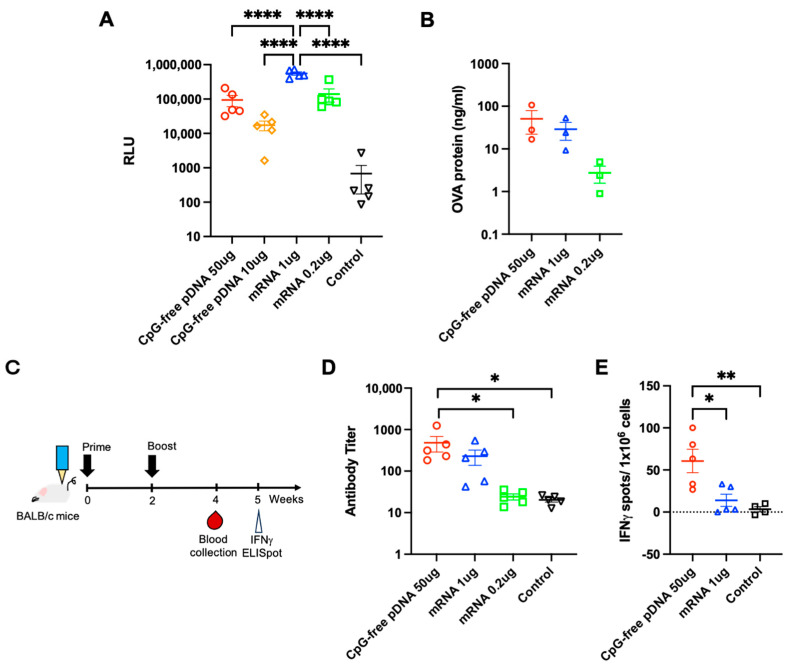
Comparison of PJI-delivered pDNA and mRNA vaccines on OVA expression and immune response. (**A**) LUC-mRNA (0.2 or 1 µg/20 µL) or CpG-free pDNA encoding LUC (10 and 50 µg/20 µL) were injected intradermally into C57BL/6NJcl mouse backs by PJI (*n* = 5 in each group), and luciferase activity in the injected skin tissues was analyzed 3, 6, and 24 h after the injection. Luciferase activity is represented by RLU. (**B**) OVA protein expression 24 h after the intradermal injection of the OVA-encoding CpG-free pDNA (50 µg/20 µL) or mRNA (0.2 or 1 µg/20 µL) into the BALB/c mouse skin (*n* = 3 in each group). (**C**) Time course of the experiment. The BALB/c mice were vaccinated twice (prime and boost) intradermally using PJI at a two-week interval and the anti-OVA antibody titer was analyzed at four weeks and IFN-γ ELISpot at five weeks. (**D**) Anti-OVA antibody titer at four weeks after the intradermal prime/boost injections of OVA pDNA (50 µg/20 µL) or mRNA (0.2 or 1 µg/20 µL) into the BALB/c mouse or non-vaccinated control mouse skin by PJI (*n* = 5 in each group). (**E**) OVA-specific IFN-γ-secreting splenocytes at five weeks after intradermal prime/boost injections of OVA pDNA (50 µg/20 µL) or mRNA (1 µg/20 µL) into the BALB/c mouse skin by PJI or non-vaccinated mice (*n* = 5 in each group). All the results are shown as the mean ± SEM. The *p*-values were analyzed using the one-way ANOVA test adjusted for multiple testing using Tukey’s multiple comparisons test. * *p* < 0.05, ** *p* < 0.01, **** *p* < 0.0001.

To compare mRNA and pDNA delivery into the skin by PJI, instead of comparing equivalent doses of mRNA and pDNA, we explored using mRNA and pDNA doses to achieve similar levels of antigen expression, which would provide a more effective starting point for the comparison of vaccine-induced immune responses. LUC-mRNA (0.2 and 1 µg) and CpG-free pDNA encoding LUC (pCpGfree-LUC; 10 and 50 µg) were injected intradermally into the backs of the C57BL/6NJcl mice, and the injected skin was harvested 24 h later and analyzed for luciferase activity. The luciferase activity of 0.2 µg LUC-mRNA was approximately the same as that of the 50 µg CpG-free pDNA injection (Figure 3A). In another expression model using OVA-encoding CpG-free pDNA (50 µg) and mRNA (0.2 and 1 µg), the OVA expression levels were more comparable between 50 µg CpG-free pDNA and 1 µg mRNA (Figure 3B). These results strongly indicate that naked mRNA vaccines require a smaller effective dose of nucleic acid-based vaccines (50–250 times less) than pDNA vaccines via PJI to achieve approximately equivalent antigen expression. Next, to compare vaccine-induced immune responses between CpG-free pDNA and mRNA, the BALB/c mice were intradermally vaccinated twice at a two-week interval by PJI and then analyzed for serum anti-OVA antibody titer at four weeks and IFN-γ-ELISpot at five weeks (Figure 3C). The CpG-free pDNA (50 µg) vaccination induced the highest overall anti-OVA antibody titer, 2.1-fold compared to 1 µg mRNA, while 0.2 µg mRNA barely produced any antibody titer above the non-vaccinated negative control group (Figure 3D). The CpG-free pDNA (50 µg) also induced a significant increase in OVA-specific IFN-γ-secreting splenocytes compared with mRNA (1 µg) (Figure 3E). The pDNA delivered via PJI can efficiently induce antibody production and cell-mediated IFN-γ expression during the immune response. In comparison, the PJI-delivered mRNA elicited a more limited immune response, despite similar levels of antigen expression.

### 3.4. Pro-Inflammatory Factors at the Injection Site and Lymph Nodes

**Figure 4:**
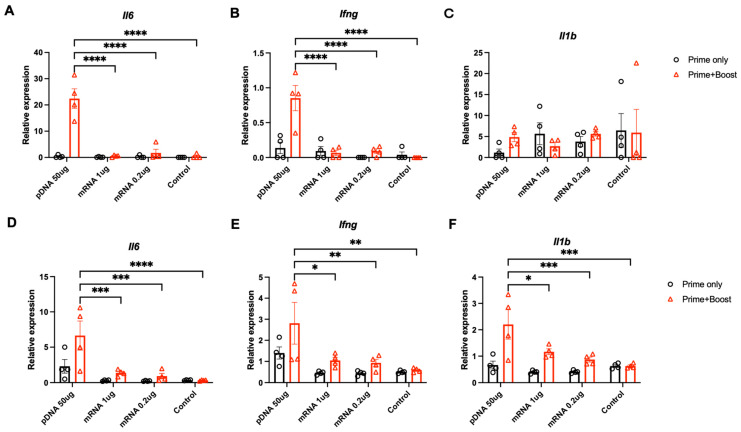
Comparison of PJI-delivered pDNA and mRNA vaccines on pro-inflammatory factors at the injection site and LN. Relative mRNA expression of inflammatory factors (**A**) IL-6 (*Il6*), (**B**) IFN-γ (*Ifng*), and (**C**) IL-1β (*Il1b*) in the injected skin tissues, and (**D**) IL-6 (*Il6*), (**E**) IFN-γ (*Ifng*), and (**F**) IL-1β (*Il1b*) in the proximal LNs measured by quantitative real-time PCR, at 24 h after the intradermal prime (prime only) or prime/boost (prime/boost) injections of OVA-encoding CpG-free pDNA (50 µg/20 µL), mRNA (0.2 or 1 µg/20 µL), or PBS (control) in BALB/c mice skin (*n* = 4 in each group). The relative expression of each cytokine was normalized to that of *β-actin*. All the results are shown as mean ± SEM. The *p*-values were analyzed using the two-way ANOVA test with Tukey’s multiple comparison test. * *p* < 0.05, ** *p* < 0.01, *** *p* < 0.001, **** *p* < 0.0001.

In addition to adaptive immune responses, previous studies have indicated that pDNA and mRNA vaccines may also affect inflammation or innate immune responses differently owing to their specific receptors and signaling pathways [27]. To investigate potential pro-inflammatory differences between pDNA and mRNA vaccines, we compared the changes in inflammatory factors in the injected skin regions and proximal lymph nodes (LNs) after prime-only and prime/boost injections using PJI. In the skin, the pDNA vaccine significantly increased IL-6 and IFN-γ after the prime/boost injections, whereas the mRNA vaccine induced an increase in IL-1β earlier, at 24 h after singular prime injection, suggesting distinct nucleic acid-based differences in activated immune mechanisms at primary injection sites (Figure 4A–C). In the proximal LNs, the pDNA vaccine elicited the strong upregulation of all three inflammatory factors after the prime/boost injections, whereas the mRNA vaccine induced weaker responses overall (Figure 4D,F), in line with the observed lower overall immunogenicity of mRNA to stimulate adaptive immune responses.

### 3.5. Comparison of mRNA Delivered by PJI and in Combination with LNP

**Figure 5:**
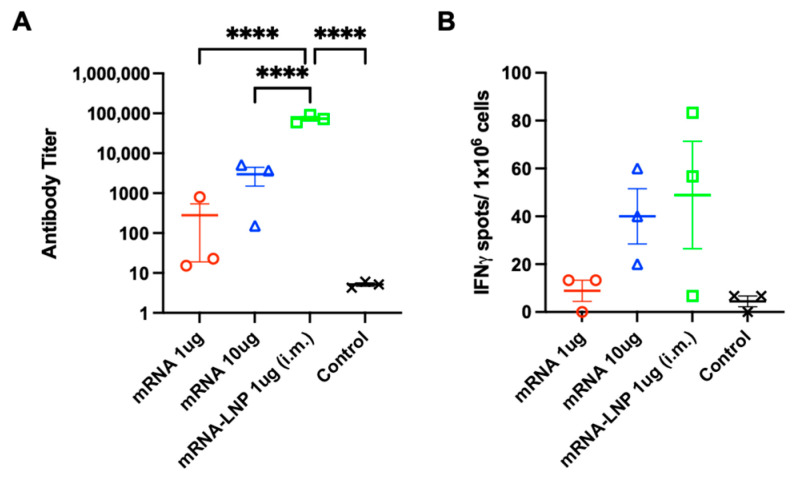
Comparison of mRNA delivered by PJI and in combination with LNP (i.m.). (**A**) Anti-OVA antibody titer at four weeks after the prime/boost injections of OVA mRNA (1 or 10 µg/20 µL) into mouse skin by PJI, intramuscularly administered OVA mRNA in combination with LNP vaccine (mRNA-LNP 1 µg/40 µL (i.m.)) into mouse thigh muscles by needle syringe, or non-vaccinated (control) mice (*n* = 3 in each group). (**B**) OVA-specific IFN-γ-secreting splenocytes at five weeks after the prime/boost injections of OVA mRNA (1 or 10 µg/20 µL) into mouse skin by PJI, or intramuscularly administered OVA mRNA-LNP (1 µg/40 µL (i.m.)) into mouse thigh muscles by needle syringe, or non-vaccinated mice (*n* = 3 in each group). All the results are shown as the mean ± SEM. The *p*-values were analyzed using the one-way ANOVA test adjusted for multiple testing using Tukey’s multiple comparisons test. **** *p* < 0.0001.

Given the limited ability of the PJI-delivered naked mRNA to stimulate both antibody- and cell-mediated IFN-γ immune responses, we compared the effects of an intradermal PJI-delivered mRNA vaccine by increasing the mRNA dose (from 1 to 10 µg). We added a positive control vaccination group using an intramuscular needle-administered mRNA vaccine in combination with LNP containing 1 µg of mRNA (mRNA-LNP (i.m.)) for comparison (Figure 5A,B). Increasing the mRNA dose from 1 µg to 10 µg improved the anti-OVA antibody titer (by 10.7-fold), but it was still greatly eclipsed by mRNA-LNP (i.m.) vaccination (by 265-fold compared to mRNA 1 µg; Figure 5A). Regarding cell-mediated immune stimulation, the higher-dose mRNA vaccine induced a 4.5-fold increase in the OVA-specific IFN-γ-secreting T cell response, whereas the mRNA-LNP (i.m.) vaccine induced a 5.5-fold increase (Figure 5B). These results show that although increasing the mRNA dose can make the naked mRNA vaccine more effective, the mRNA-LNP (i.m.) vaccine was still more efficient than the PJI-delivered naked mRNA vaccine in eliciting effective antibody and cell-mediated immune responses, which may provide potential variables to improve mRNA vaccines using PJI.

## 4. Discussion

During the development of mRNA therapy, the first evidence of protein expression following in vivo mRNA delivery was observed in 1990 [28]. The mechanism of innate immune activation by mRNA has been elucidated, in which mRNA is recognized by Toll-like receptor (TLR)-3, TLR-7, and TLR-8, resulting in type I interferon production. As this innate immune activation may suppress protein expression from the mRNA itself [29], the modification of uridine nucleotides in the mRNA with pseudouridine prevents recognition by TLR-7 and TLR-8, thereby suppressing innate immune activation and enhancing protein expression [30]. pDNA has been reported to be recognized by TLR-9 or other DNA sensors to activate innate immunity, but is commonly used unmodified despite its similar beneficial potential in avoiding undesirable innate immune activation. In this study, CpG-free pDNA induced higher antigen expression than regular CpG-containing pDNA. This could be due to its stronger hEF-1a promoter, and the absence of CpG motifs means potentially less stimulation of innate immune receptors, such as TLR-9. Our results showed that CpG pDNA was weakly immunogenic overall. The CpG-free pDNA showed a stronger innate immune activation effect in mice, which corresponded to stronger humoral and cell-mediated adaptive immune responses, but required a higher pDNA dose to fully express antigens due to its weak protein expression capacity compared to mRNA. Therefore, DNA modifications that control innate immune activity may be beneficial in improving DNA vaccine efficiency.

In the early stages of DNA vaccine development, DNA vaccines were mostly administrated via the intramuscular route [31,32]. Later, several studies reported that the intradermal delivery of DNA vaccine induced a stronger immunogenic response compared to intramuscular delivery, which was attributed to the enrichment of dendritic cells in dermal skin [33,34,35]. Based on this premise, a number of COVID-19 vaccine candidates in development were intradermal DNA vaccines [16,17,18,36,37]. To compare humoral response efficacy between the intramuscular route and intradermal route, we have previously reported that PJI-injected DNA vaccine could induce a more robust antibody production in rats compared to intramuscular administration, even though the concentration of pDNA was lower in the former group [16]. These findings indicate that the intradermal route may be the mainstream method for DNA vaccine administration in the future.

Besides the administration route, improvement in pDNA and mRNA delivery technology is also an important issue in vaccine development because they need to be taken up into cells for the efficient induction of expression. In human clinical trials of DNA vaccines developed during the COVID-19 pandemic, namely, INO-4800 [9], AG0302 [8], and ZyCoV-D [7], electroporation or needleless injectors were used for intradermal administration. In a functional SARS-CoV-2 DNA vaccine model in mice, we previously showed that intradermal administration using PJI without adjuvants induced high antibody titers and T cell immune responses by achieving high antigen expression [17]. In this study, antibody production without an adjuvant was also confirmed by the intradermal administration of pDNA and mRNA using a needle-free injector. However, the immune response was not as strong as that induced by the intramuscular administration of the LNP-encapsulated mRNA (Supplementary Figure S1). These results suggest that the development of a simple vaccine formulation with nucleic acid drugs may be difficult to achieve an optimum balance between enhanced antigen expression and innate immune activation.

LNPs are one of the most commonly used components in mRNA vaccines [26]. LNPs are synthesized using cationic lipids, polyethylene glycol, cholesterol, and phospholipids. mRNA encapsulation within LNPs provides stability and cell permeability to the mRNA. Encapsulated mRNA is rapidly released from the endosomes after cellular uptake to induce antigen expression. In addition, LNPs may act as adjuvants to stimulate pro-vaccine innate immune responses. Improvements in LNPs for innate immune activation and mRNA delivery technologies have enabled the development of mRNA vaccines. However, owing to the strong activation of the immune response, the mRNA-LNP vaccine formulation also commonly presents with relatively strong side effects, such as fever or general malaise, which may be considered an adverse reaction due to heightened innate immune activation by LNP. If the specialized features of the injection device-based nucleic acid vaccine can be maximized to develop a formulation that achieves high antigen expression while reducing the adverse reactions of LNP, an ideal vaccine formulation can be developed with high efficacy and low adverse reactions. Therefore, there may be potential for developing mRNA vaccine formulations tailored for injector delivery as opposed to the intramuscular route, such as with LNPs or other suitable adjuvants.

The temperature stability of the mRNA-LNP formulation is also a known problem, requiring an ultra-low-temperature freezer and special transportation methods to maintain the quality of the formulation. This limits its supply to economically less-developed and climate- or geographically challenged areas [38]. Therefore, the development of technologies for more stable vaccine formulations and modalities is urgently required. DNA vaccines have not yet attained the high efficacy of mRNA vaccines, but their stability is very high; the quality retention period at 25 °C is 2–12 h for approved mRNA vaccines, whereas DNA vaccines are stable for up to three months [39]. If a more temperature-stable and effective DNA vaccine can be developed in conjunction with efficient injectors, these vaccines may become more viable worldwide, minimalizing the requirement for specialized vaccine storage, providing affordable access to developing countries. Needle-free injectors are a promising technology for future development as they may help reduce accidental medical injuries involving needles.

## 5. Conclusions

In conclusion, we have shown that PJI can deliver simple DNA and mRNA vaccines intradermally into mouse skin. Compared to mRNA vaccines, modified pDNA vaccines strongly elicited antigen-specific antibodies, cell-mediated immune responses, and pro-inflammatory cytokines. Further studies are required to refine the use of mRNA vaccines using PJI, including the potential use of LNP or other adjuvants, to induce a more effective immune response in mice.

## Data Availability

The data presented in this study are available from the corresponding author upon reasonable request.

## Supplementary Materials

The following supporting information can be downloaded at: https://www.mdpi.com/article/10.3390/vaccines12070757/s1, Figure S1: Summary of immune responses mediated by DNA and mRNA vaccines delivered via PJI.
