# Supplementary material for: Immunogenic Comparison of Nucleic Acid-Based Vaccines Administered by Pyro-Drive Jet Injector
Source: Vaccines (Basel). 2024 Jul 9;12(7):757. doi: 10.3390/vaccines12070757 (PMC11281653; doi:10.3390/vaccines12070757)
Supplement: Supplementary file 1 [file vaccines-12-00757-s001.zip › vaccines-3056669-supplementary.pdf]

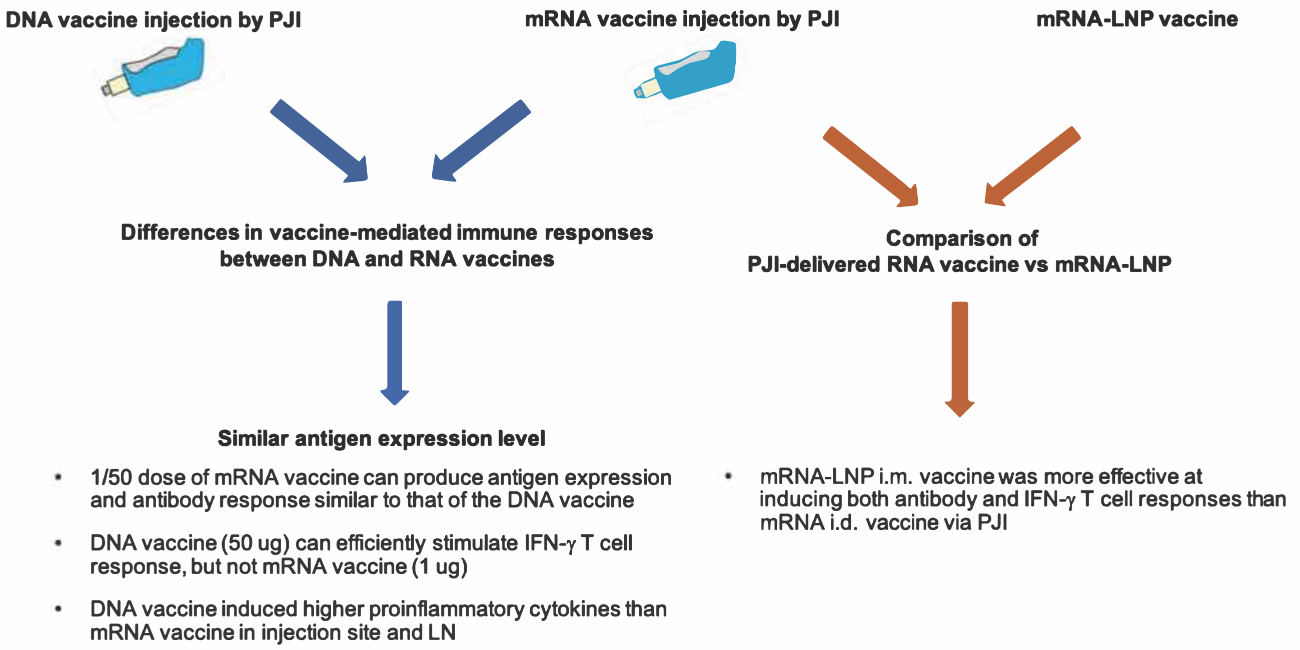

Supplementary Figure S1. Summary of immune responses mediated by DNA and mRNA vaccines delivered via PJI.
